# A deterministic method for estimating free energy genetic network landscapes with applications to cell commitment and reprogramming paths

**DOI:** 10.1098/rsos.160765

**Published:** 2017-06-07

**Authors:** Victor Olariu, Erica Manesso, Carsten Peterson

**Affiliations:** 1 Computational Biology and Biological Physics, Department of Astronomy and Theoretical Physics, Lund University, Lund 22362, Sweden; 2 Center for Models of Life, Niels Bohr Institute, University of Copenhagen, Copenhagen 2100, Denmark

**Keywords:** energy landscape, deterministic models, stem cell commitment, reprogramming

## Abstract

Depicting developmental processes as movements in free energy genetic landscapes is an illustrative tool. However, exploring such landscapes to obtain quantitative or even qualitative predictions is hampered by the lack of free energy functions corresponding to the biochemical Michaelis–Menten or Hill rate equations for the dynamics. Being armed with energy landscapes defined by a network and its interactions would open up the possibility of swiftly identifying cell states and computing optimal paths, including those of cell reprogramming, thereby avoiding exhaustive trial-and-error simulations with rate equations for different parameter sets. It turns out that sigmoidal rate equations do have approximate free energy associations. With this replacement of rate equations, we develop a deterministic method for estimating the free energy surfaces of systems of interacting genes at different noise levels or temperatures. Once such free energy landscape estimates have been established, we adapt a shortest path algorithm to determine optimal routes in the landscapes. We explore the method on three circuits for haematopoiesis and embryonic stem cell development for commitment and reprogramming scenarios and illustrate how the method can be used to determine sequential steps for onsets of external factors, essential for efficient reprogramming.

## 1. Introduction

Enforced guiding of developmental processes including those of cellular reprogramming could benefit from *in silico* dynamical modelling by tuning parameters for protein concentrations and other factors involved in the rate equations describing the systems. However, exhaustive scanning of different concentrations of such factors is not practical in a rate equation setting. A more profitable approach would be to map out the corresponding free energy landscape. The latter concept goes back to the Waddington landscape metaphor, which is frequently used to qualitatively visualize developmental processes such as stem cell commitment and reprogramming (e.g. [1]). The underlying idea is that the dynamics of biochemical equations, governing a specific developmental process, can be represented as movements in a free energy landscape such that lineage choices are paths between stable cell states. This notion is based upon a potential correspondence between solving the equations of motion and minimizing the corresponding free energy. While this relationship is often true in physics models, a quantitative relationship between the biochemical dynamics and the free energy landscape has not been widely exploited in developmental processes. This is due to the fact that the frequently used Michaelis–Menten or Hill kinetics do not have a corresponding free energy from which the rate equations are given by a gradient. For this reason, different approaches to approximate the energy landscape have been explored for small systems. In Wang *et al.* [2], a stochastic method is exploited where the dynamical equations provide probability distributions from which the free energies are estimated from the logarithms. This approach becomes very time-consuming when the network includes many genes. In Bhattacharya *et al.* [3] and Zhou *et al.* [4] quasi-potential methods based upon Lyapunov theory are developed where the energy or potential is decomposed into two terms: one related to the dynamical equations and the other chosen to minimize its effect on state transitions.

An approach to efficiently map molecular dynamics onto a free energy landscape is of value, far beyond illustrative purposes and theoretical curiosity. With proper search strategies, it enables finding optimal paths between cellular states (or basins of attraction).

Here we devise a strategy wherein Hill functions are replaced by sigmoids. The latter can be associated with approximate free energy functions, which allow for a rapid deterministic estimate of all free energy values in a dense and high-dimensional grid. Sigmoids are very good approximations to Hill function kinetics, in particular, when cooperativity is involved, which is often the case in transcriptional processes. Furthermore, this formulation allows for exploring different temperatures deterministically, thereby tuning to different average noise levels. We then map the determined free energy landscape into a graph, compute all the possible stable states or attractors. Finally, we calculate the shortest path between two of any stable states using the Dijkstra algorithm [5], which is well established in e.g. communication routing problems, thus providing a practical means to determine optimal paths for both cell commitment and reprogramming.

In brief, our method consists of the following steps:

1. Given time-series data for expression and binding data for key genes, determine the corresponding parameters for rate equation models. In cases where time-series data are not available, we use parameters that give rise to known/assumed steady states. In our case, the rate equation models are based upon sigmoids rather than the commonly used Hill functions. Apart from this replacement, the procedure for this step is standard and can include bifurcation and sensitivity analysis.
2. With sigmoidal gain functions, the fitted parameters then directly estimate the free energy functions, which then ‘summarize’ the dynamics for different gene/protein concentration values.
3. Being armed with these determined energy functions (or surfaces), which are discretized into a grid, enables us to determine what it takes to move from one state to another in terms of changing concentrations subject to different conditions, e.g. following the shortest path.

It should be emphasized that the focus of our approach is state transitions caused by external cues—hence deterministic methods are appropriate. With regard to spontaneous transitions caused by few copies of the molecules giving rise to internally driven transitions, interesting approaches have recently been put forward [6,7]. It should also be noted that for gene networks with large anti-symmetry, e.g. a ring oscillator, the framework proposed in this study does not necessarily work. However, there are many important gene regulatory networks where the non-symmetry does not push the system towards an oscillatory behaviour and our framework can be successfully used. Also, our method is developed for a fixed landscape, which makes the path concept stand out. Alternatively, one can include a reprogramming force explicitly with fixed parameters into the free energy function. In this way, another landscape will emerge, where the origin in terms of attractors might get lost.

Most of what follows will concern the mapping onto free energy landscapes and the determination of shortest paths. For larger networks, we share the same challenge as everyone else—to reverse-engineer measured time series, binding strengths and over-/under-expression outcomes to a set of interactions. Undetermined interactions can in such cases be estimated from exhaustive searches with optimal fits as criteria [8]. After demonstrating how to estimate free energies relating to sigmoidal gain functions within the so-called mean field formalism and how to explore the mean free energy landscape, we illustrate our approach with two toy-model switch examples: a single self-interacting gene and two mutually repressing genes. The latter commonly occurs in many developmental systems [9–14]. We then exploit our method in two more elaborate instances from haematopoiesis involving three genes: the GATA2, GFI1 and GFI1b system [15] and the GATA1, GATA2 and PU.1 system [8], respectively. Finally, we study the four-gene network governing the embryonic stem cell switch by NANOG, OCT4-SOX2, FGF4 and a differentiation gene G [16].

This work has more the character of methods development than providing deeper understanding of the biology systems probed. However, for the embryonic stem cell system we do put forward a reprogramming recipe example.

## 2. Results

After claiming that Hill functions and sigmoids have similar shapes for fitting to time-series data, we demonstrate how the latter can be derived from approximate free energy functions. Subsequently, we explain how these can be used for finding cell states and paths in between. After that, sigmoids and the corresponding free energy functions are employed to analyse five example networks, where three originate from the real world.

### 2.1. Hill functions versus sigmoids

**Figure 1.**
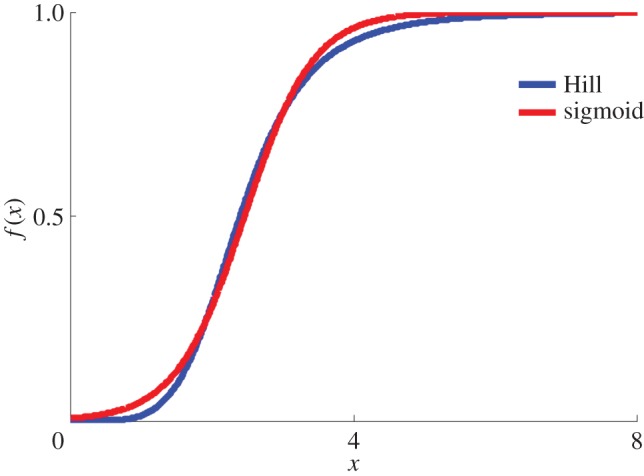
A typical comparison between a Hill and a sigmoidal gain function with parameters *k*=2.4, *n*=5 and *a*=2 and *c*=2.4 (see text).

Hill gain functions, *h*(*x*)=*x*^*n*^/(*k*^*n*^+*x*^*n*^), are commonly used in dynamical rate equations for transcriptional processes. With cooperativity (*n*>1), these are almost indistinguishable from sigmoidal ones, *g*(*x*)=1/(1+*e*^−*a*(*x*−*c*)^), for proper choice of parameters. This is not surprising—the only difference is power laws versus exponentials in the denominators. An example is shown in figure 1. Note that using sigmoids rather than the Hill function represents no difference when it comes to employing established tools like bistability analysis. There is no free energy function *F* from which the Hill function *h*(*x*) can be derived by taking the gradient of *F*. This is in contrast to the case for sigmoidal functions *g*(*x*) as we demonstrate below, where we start out from an approximate free energy function *F* and from there derive a sigmoid as a gradient. In one early rate equation approach to transcriptional dynamics, sigmoids were actually used [17].

### 2.2. Sigmoids and free energies

For simplicity and illustration purposes, we first consider two mutually interacting genes with activations *v*_1_ and *v*_2_, respectively, interaction strengths *ω*_*ij*_ and external forces *l*_*i*_ with a free energy *F*(*v*) given by

2.1 F(v)=E(v)−TS(v),

where the two terms representing internal energy and entropy are

2.2 $$E(v)=-\frac{1}{2}(\omega_{12}v_{1}v_{2}+\omega_{21}v_{2}v_{1}+l_{1}v_{1}+l_{2}v_{2})$$

and

2.3 $$S(v)=-\frac{1}{2}\sum_{i}[v_{i}\log v_{i}+(1-v_{i})\log(1-v_{i})],$$

respectively. Here *T* is the temperature or noise level. Steady-state solutions for each gene *i* are then obtained by taking the partial derivative of equation (2.1) with respect to *v*_*i*_ and setting it to zero,

2.4 $$\frac{\partial F(v)}{\partial v_{i}}=0,$$

yielding

2.5 $$\begin{array}{rl} v_{1} & =g\left(-\frac{1}{2T}\frac{\partial E(v)}{\partial v_{1}}\right)=g\left(-\frac{1}{2T}(-\omega_{12}v_{2}-\omega_{21}v_{2}-l_{1})\right)\\ \text{and}\;\;v_{2} & =g\left(-\frac{1}{2T}\frac{\partial E(v)}{\partial v_{2}}\right)=g\left(-\frac{1}{2T}(-\omega_{21}v_{1}-\omega_{12}v_{1}-l_{2})\right), \end{array}\}$$

where *g*(⋅) is the sigmoid function, *g*(*x*)=1/(1+*e*^−*x*^). In the noiseless limit (T→0), a binary system is obtained. For symmetric interactions, these equations are correct. However, the interaction matrix with elements *ω*_*ij*_ is often unsymmetrical for biological networks, in which case equations (2.5) should read

2.6 $$\begin{array}{rl} v_{1} & =g\left(-\frac{1}{2T}(-\omega_{21}v_{2}-l_{1})\right)\\ \text{and}\;\;v_{2} & =g\left(-\frac{1}{2T}(-\omega_{12}v_{1}-l_{2})\right), \end{array}\}$$

where *ω*_21_*v*_2_ and *ω*_12_*v*_1_ are the forces acting upon *v*_1_ and *v*_2_, respectively. Unfortunately, equations (2.6) can, in this unsymmetric case, not be derived from the free energy of equation (2.3) as it stands. Therefore, we propose an approximation by decomposing the free energy into separate parts, each of which having its dynamical variables with regard to taking the gradient

2.7 $$E(v_{1},v_{2})=E_{1}(v_{1}|v_{2})+E_{2}(v_{2}|v_{1})=-\frac{1}{2}(\omega_{21}{\text{v}}_{\text{1}}v_{2}+l_{1}{\text{v}}_{\text{1}})-\frac{1}{2}(\omega_{12}{\text{v}}_{\text{2}}v_{1}+l_{2}{\text{v}}_{\text{2}}),$$

where **v**_**1**_ and **v**_**2**_ represent the active variables in the decomposition, which are subject to the gradient leading to equation (2.6), whereas *v*_1_ and *v*_2_ are merely parts of acting forces together with *ω*_*ij*_. This decomposition is the key approximation in this work. The entropy part (equation (2.3)) remains unchanged and is crucial for establishing the sigmoidal behaviour. For practical purposes, one then simply integrates the rate equations given active interactions to obtain the different parts of equation (2.7). Throughout this work, we will suppress the notational specification of active genes as it naturally follows from such integrations.

Applying the Euler approach to finding the steady state (equation (2.6)) leads to

2.8 $$\eta \frac{dv_{i}(t)}{dt}=g\left(-\frac{1}{2T}(-\omega_{ji}v_{j}(t)-l_{i})\right)-v_{i}(t),$$

with *η* being a step parameter. In equation (2.8), the sigmoid function is the counterpart of Hill kinetics interpreting the last term with degradation. Hence, we have identified a mathematical form for transcriptional dynamics, the sigmoid, that can be derived from an approximate free energy function. Sigmoidal representation of the dynamics thus leads to having an energy landscape at our disposal. The relationship between the free energy and the corresponding rate equations originates from the so-called mean field approximation in spin physics (see Methods section). Within our approximation, we only exploit this relation on a gene-by-gene basis.

For more than two genes, equation (2.2) generalizes to

2.9 $$E(v)=-\frac{1}{2}\sum_{ij}(\omega_{ij}v_{i}v_{j}+l_{i}v_{i}),$$

where the indices *i* and *j* are appropriately summed over and a decomposition *E*=*E*_1_+*E*_1_+⋯*E*_*N*_ is to be understood for the different genes *i* and *j*, as in equation (2.7).

Equation (2.8) is then generalized to

2.10 $$\eta \frac{dv_{i}(t)}{dt}=g\left(-\frac{1}{2T}\left(-\sum_{j}\omega_{ji}v_{j}(t)-l_{i}\right)\right)-v_{i}(t).$$

The energy landscape is easily estimated on a grid at a desired resolution of the gene expression values *v*_*i*_ (see below). The approximate *F*(*v*) provides free energy for all gene expression values and not just for the steady states or attractors.

Equations (2.1)–(2.10) are well known from the mean field approximation in spin physics, and can be easily generalized to more interacting genes (Methods)—higher polynomials in equation (2.9)—again with single-gene decomposition dynamics.

### 2.3. Exploring the free energy landscape

The free energy (equation (2.1)) is given for continuous gene expression values. It is preferable for practical reasons to discretize the latter into a fixed number of bins. The landscape then turns into a graph where the vertices represent states of the cell, given by expression values of the genes in the network. The edges between adjacent vertices are directed from vertices of high to low energy. The attractors (stable states) are then identified as vertices with only entering edges (Methods).

Experimentally, it is feasible to convert a cell fate by transcription factor-based programming and reprogramming [18–20]. In the case of programming, a tissue-specific cell can be converted to a related tissue-specific cell without transiting through the uncommitted state [18,19], while the reprogramming consists in reverting the route from a pluripotent stem cell to a terminally differentiated somatic cell [20].

Given a gene regulatory network describing differentiation processes from an uncommitted cell to various tissue-specific cell types, commitment and reprogramming can be seen as paths between two stable states of interest (i.e. attractors) in the free energy graph representation. In principle, it is not possible to move from a stable state to another, as, by definition, the vertex corresponding to an attractor does not have exiting edges (Methods). However, if the indirect version of the graph is considered, the Dijkstra algorithm [5] offers a recipe for the most efficient commitment/(re)programming process in terms of free energy changes by providing the shortest path between the two nodes representing the stable states of interest.

We explore our approach below initially with synthetic one- and two-gene examples. Then we employ the method on three- and four-gene real-world applications involving haematopoietic progenitors and embryonic stem cells. The corresponding equations together with parameter values are found in the electronic supplementary material, S1.

### 2.4. A synthetic single-gene switch

**Figure 2.**
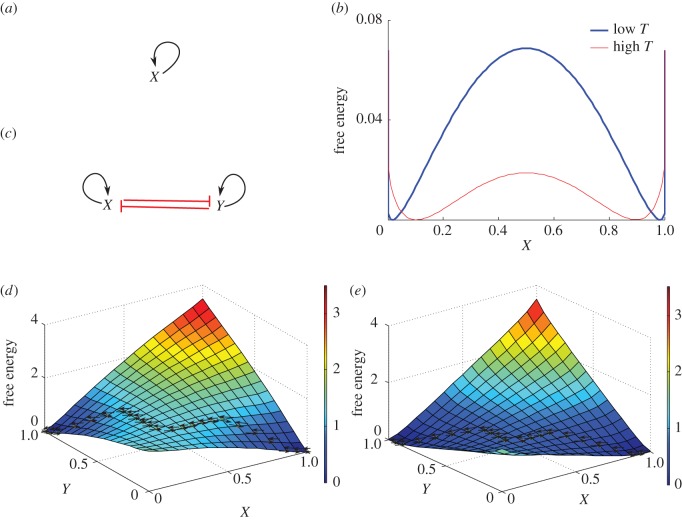
Synthetic single- and two-gene networks along with free energies at different temperatures. (*a*) A single-gene positive self-interaction network with (*b*) its free energy where blue and red show the free energies for low and high temperatures *T*, respectively. (*c*) The two gene mutual inhibition circuit with positive auto-regulations and (*d*) its free energy when *T* is low. There are two attractors with a high peak in between. The dashed arrow lines represent the shortest paths between the attractors. (*e*) The free energy when *T* is high and the peak in between the attractors is low. The same notation as in (*d*). The corresponding equations together with parameter values are found in the electronic supplementary material, S1.

A single-gene network with positive self-interaction (figure 2*a*) can exhibit switch behaviour. Being one-dimensional, it serves as an illustrative introduction for displaying the effects of noise (temperature). The free energy was calculated for low and high values of the temperature *T*. Figure 2*b* shows the free energy exhibiting two attractors at low and at high gene expression values. Varying the temperature changes the landscape dramatically. For low *T*, there is a high peak between the two attractors (blue line), whereas for high *T* the peak between the two attractors diminishes and the attractors move inwards (red line). Hence, increasing the temperature (noise level) facilitates the transition from one attractor to another. Furthermore, we conducted a sensitivity analysis to variations of the degradation rate, self-interactions and external signal strengths showing that these parameters also have an important impact on the landscape (electronic supplementary material, S1).

### 2.5. Two mutually repressing genes: the paradigmatic switch

Network motifs with two mutual inhibiting transcription factors and positive self-interactions (figure 2*c*) play important roles in stem cell fate decisions. Examples are NANOG/GATA6 in the endoderm [9,21] and OCT4/CDX2 in the trophectoderm [10,22–25] lineage choices, PU.1/GATA1 in haematopoiesis [12–14] and NKX6/PTF1a in pancreatic progenitor cells [11].

We calculate the free energy for the motif in figure 2*c* denoting the two genes by *X* and *Y* , respectively. Models with this topology often exhibit bistability. The free energy displays two low-energy areas (figure 2*d*,*e*): the blue areas where *X* and *Y* expressions are high and low (*X*^*H*^, *Y* ^*L*^) and *vice versa* are the basins of attraction of the two stable states. These represent two alternative stem cell fates. The circuit converges towards either one or the other depending on: (i) the basin of attraction of the initial state, (ii) the direction of the dynamics imposed by external signals, and (iii) the noise level (*T*) in the system. Figure 2*d*,*e* shows the effect of varying *T* on the free energy. At low *T*, the free energy exhibits a high protuberance on the level surface between the two basins of attraction (figure 2*d*). At high *T*, the barrier between the two attractors diminishes, facilitating the transition between the two states (figure 2*e*).

Once the free energy was mapped onto a graph, we identified the two attractors and applied the Dijkstra algorithm [5] to compute the shortest path between them. Figure 2*d*,*e* shows the best strategies for switching between the two cell fates for both low and high temperatures. The optimal path from the (*X*^*H*^, *Y* ^*L*^) state to the (*X*^*L*^, *Y* ^*H*^) state is shown by dashed arrows. Initially, the highly expressed gene needs to decrease expression to a value low enough for the other gene to start being expressed. Next, the newly expressed gene increases its expression value until its maximum, while the other gene slowly decreases towards no expression—the cell is now in the new state. The temperature affects the optimal path between the two attractors. At low *T*, a higher number of initial steps are required for escaping the basin of attraction and approaching the switch area—the hump between attractors (figure 2*d*). At high *T*, the departure from the original basin of attraction and the climb towards the switch area occurs more swiftly (figure 2*e*).

### 2.6. The GATA2-GFI1-GFI1B network in myelo-lymphoid lineage decisions

**Figure 3.**
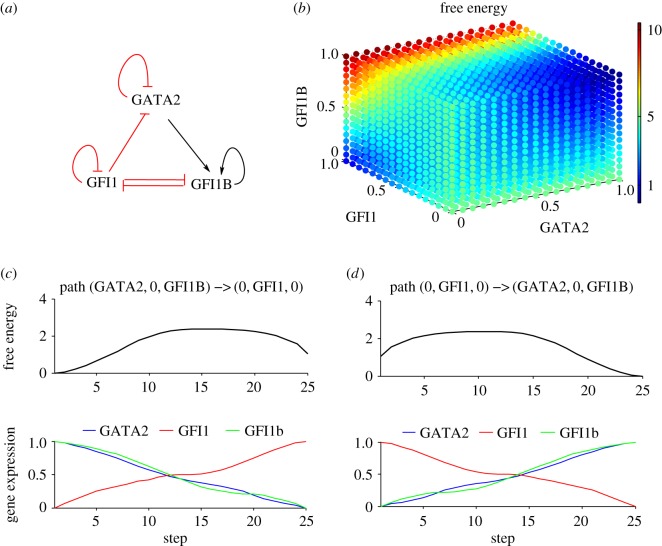
The GATA2-GFI1-GFI1B network and the corresponding free energy and shortest paths between the attractors. (*a*) The corresponding circuit with the GFI1B and GFI1 mutual inhibition, GFI1 repressing GATA2 and GATA2 inducing GFI1B. GATA2 and GFI1 self-interact negatively while GFI1B is characterized by positive auto-regulation. (*b*) The free energy exhibits two attractors: (1) GATA2/GFI1B-high and GFI1-low and (2) GFI1-high and GATA2/GFI1B-low, respectively. The corresponding equations together with parameter values are found in the electronic supplementary material, S1. (*c*) Variation of the free energy and gene expressions along the shortest path between (1) and (2). (*d*) The corresponding variation along the shortest path between (2) and (1).

This three-gene network was recently proposed [15] to govern the entry into the myelo-lymphoid lineages, where GATA2 may function in a regulatory loop to modulate GFI1-GFI1b cross-antagonism. In other studies, it was shown that GFI1b is subject to positive auto-regulation [26,27], while GFI1 and GATA2 are auto-repressive [8,28]. We unified these results in the circuit shown in figure 3*a*.

We computed the free energy, which exhibits two stable states. The first one is where the expressions of GFI1B and GATA2 are high while GFI1 is low—the cell is in a haematopoietic stem cell (HSC) state. The second attractor is the state where GFI1 is high and GATA2 and GFI1B are low—the cell is committed to the myelo-lymphoid lineage (figure 3*b*).

The optimal commitment path of HSC towards the myelo-lymphoid lineage is described by the calculated best pathway between the first and second attractor (figure 3*c*). The most favourable commitment dynamics involve GATA2 and GFI1B expressions decrease while GFI1 starts to be expressed; the cell commits to the myelo-lymphoid lineage. The optimal strategy for reprogramming of a cell committed to the myelo-lymphoid lineages back to the HSC-like state is shown in figure 3*d*. Initially, the GFI1 expression has to decrease to lower level followed by increase in expression of GFI1B and GATA2. The switch between the two cell states corresponds to the jump over the high peak in the free energy.

### 2.7. The GATA2-GATA1-PU.1 network in erythroid lineage decisions

**Figure 4.**
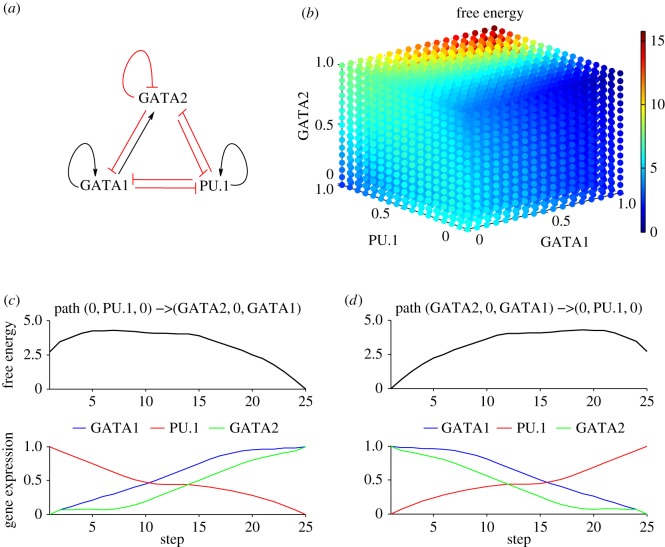
The GATA2-GATA1-PU.1 network and the corresponding free energy and the shortest paths between the attractors. (*a*) The GATA2-GATA1-PU.1 transcription factor circuit with the well-established GATA1-PU.1 mutual inhibition and positive auto-regulations, GATA1 repressing GATA2 while GATA2 induces GATA1. The circuit includes recently discovered interactions GATA2-PU.1 mutual inhibition and GATA2 negative self-interaction. (*b*) The free energy exhibiting two attractors: (1) GATA2, GATA1-high and PU.1-low and (2) PU.1-high and GATA2, GATA1-low. The corresponding equations together with parameter values are found in the electronic supplementary material, S1. (*c*) Variation of the free energy and gene expressions along the shortest path between (2) and (1). (*d*) The corresponding variation along the shortest path between (1) and (2).

This gene triad is known to play an important role in erythroid lineage commitment. In [8], the nature of regulatory interactions between GATA1, GATA2 and PU.1 transcription factors was inferred through erythroid differentiation microarray measurements and ChIP-Seq data. The circuit for the auto- and cross-regulatory interactions between these transcription factors in figure 4*a* was shown to best reproduce expression profiles of the network components during erythroid differentiation.

The GATA1-GATA2-PU.1 free energy displays two low-energy areas corresponding to two distinct cell states (figure 4*b*). The first one is characterized by high expression of GATA1 and GATA2 and low expression of PU.1 and corresponds to the erythroid fate choice. The second stable state, where PU.1 is highly expressed while the GATA factors are low, represents the myeloid cell fate.

We computed the shortest path between the two stable states for identifying the optimal procedures for switching the cell fate from myeloid towards erythroid and *vice versa*. Departing from a highly expressed PU.1 cell state is possible only by first increasing the GATA factors expressions. PU.1 expression level gets to low levels only when GATA2 expression is high (figure 4*c*). This result is in accordance with the conclusion in [8] that the GATA2-PU.1 negative interaction is very important for haematopoietic lineage decisions. Figure 4*d* shows the optimal path for a transition from the state where GATA factors are expressed and PU.1 is low towards a state where PU.1 is high while the other factors are low. Initially, a decay of GATA2 and an increase of PU.1 has to occur for an optimal transition towards myeloid fate. A decrease of GATA1 is necessary immediately afterwards for proceeding along the optimal path, due to antagonistic and auto-regulatory interactions of GATA1 and PU.1 [29].

### 2.8. A gene regulatory network for the embryonic stem cell fate decision

**Figure 5.**
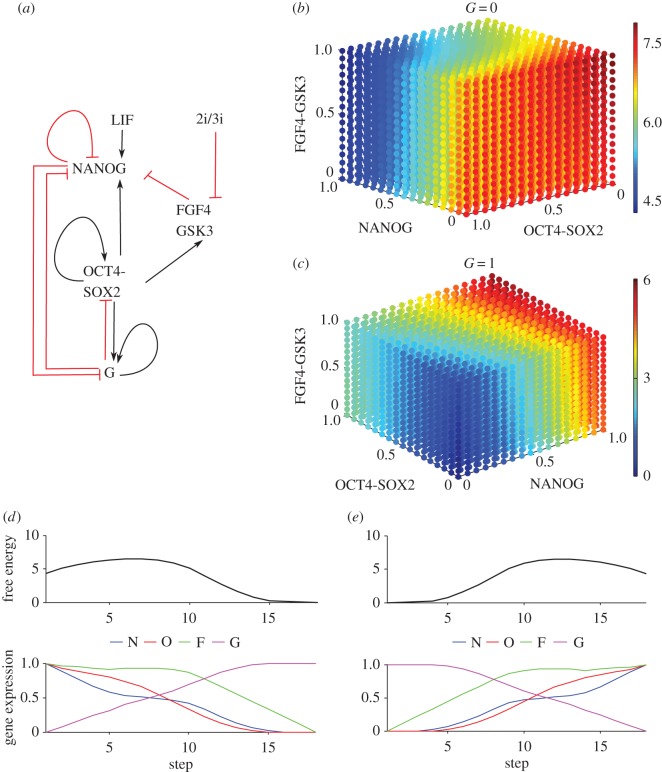
The core decision network in mouse embryonic stem cells along with the corresponding free energy and shortest paths between the attractors. (*a*) This transcription factor circuit is characterized by: OCT4-SOX2 induces NANOG, which self-interacts negatively. NANOG represses the positively self-interacting G. OCT4-SOX2 induces G suppressing both NANOG and OCT4-SOX2. LIF induces NANOG and OCT4-SOX2 induces FGF4, which suppresses NANOG. The 2i/3i medium suppresses FGF4. (*b*) The free energy shows the attractor (1) when the differentiation gene G is at low value, while NANOG, OCT4-SOX2 and FGF4 are highly expressed. The corresponding equations together with parameter values are found in the electronic supplementary material, S1. (*c*) The free energy shows the attractor (2) where G is high, while NANOG, OCT4-SOX2 and FGF4 are low. (*d*) The variation of the free energy and of the gene expressions along the shortest paths between (1) and (2). (*e*) The variation of the free energy and of gene expression along the shortest paths between (2) and (1).

Deterministic and stochastic approaches have explored the dynamics of the core regulatory network in mouse embryonic stem (mES) cells. In the deterministic approach [30], a bistable switch was identified to govern the transition between pluripotency and commitment. Stochastic models [16,31,32] explain the heterogeneity of the transcription factors and the impact of external versus internal noise on fate decisions. Here, we employ a revised version of the mES network in [16]. The OCT4/SOX2 heterodimer regulates NANOG, OCT4, SOX2 and the newly included factor FGF4. This pluripotency network (figure 5*a*) interacts with a differentiation gene G (e.g. GATA6 and SOX17) and includes the recently proposed negative NANOG self-interaction [33].

The stochastic model [16] suggests that NANOG fluctuations result from an incoherent feed-forward loop between OCT4-SOX2 and NANOG through the FGF4 node. The revised model with negative NANOG self-interaction provides an additional source of NANOG fluctuations. In both, the original and the revised, models the decision to stay in the ‘ground state’ or to commit is fundamentally stochastic.

The free energy of the core embryonic stem cell network exhibits two basins of attraction. The first attractor envisages the ‘ground state’ where NANOG and OCT4-SOX2 are highly expressed while the differentiation gene G has low expression values (figure 5*b*). The entire basin of attraction corresponds to the pluripotent stem cell state where expression of transcription factors could be heterogeneous [21,31,34–37]. The low-energy area (figure 5*b*, blue) extends on the NANOG and OCT4-SOX2 axis, showing that the cell can still be in the stem cell state while NANOG fluctuates between low and high values. The low-energy area extension towards low NANOG expression values smoothens the transitions, making the exit from the pluripotent state more likely to occur. This picture is consistent with important experimental findings: (i) the heterogeneity of NANOG expression in the embryonic stem cell state [21,31,34], (ii) the pluripotency gatekeeper role of NANOG [38]. The second stable state corresponds to a committed cell state with G highly expressed while the pluripotency factors, NANOG and OCT4-SOX2, expressions are low (figure 5*c*).

The model of the pluripotency network in figure 5*a* also hosts reprogramming from committed cells to induced pluripotent stem (iPS) cells [16] with an efficiency that peaks when OCT4 is over-expressed within a specific range as observed [39].

We explored the free energy of the model and computed the shortest paths between the two fixed points (figure 5*d*,*e*).

Figure 5*d* shows the optimal stem cell commitment path. First, NANOG expression decreases, due to fluctuations, towards low values while the differentiation gene G starts to be expressed. Next, the loss of OCT4-SOX2 expression level occurs and the expression of G rapidly increases. At the final stage, G is fully expressed, OCT4-SOX2, NANOG and FGF4 are at low levels, the cell being in a committed state.

Figure 5*e* shows the optimal reprogramming path from the somatic to the stem cell state. The recipe for optimal reprogramming proposed by our framework consists of: (i) increase the FGF pathway activity, (ii) over-express OCT4, (iii) decrease the expression of G, and (iv) NANOG must be expressed to completely down-regulate G. Our results suggest that the FGF pathway plays an important role for the reprogramming efficiency. The proposed optimal reprogramming path recapitulates the important experimental result that OCT4 over-expression is absolutely necessary for reprogramming and that NANOG is essential for the acquisition of pluripotency [38].

## 3. Discussion

We have developed a deterministic method for rapidly computing trajectories in the free energy landscape describing a gene regulatory network with some focus on cellular programming and reprogramming. This work, which uses sigmoidal functions for the underlying dynamics, is focused on methods development. The goal is to circumvent CPU-demanding stochastic simulations when mapping out energy landscapes.

To recapitulate, the key steps in our method are as follows:

— For a given model, determine the parameters by fitting equation (2.10) to measured time series if available. If this is not the case, fit to a system with the known attractor structure.
— Given these parameters, compute the free energies (equations (2.1)–(2.3) and (2.9)).
— Discretize the free energy into a grid in the space spanned by the different genes.
— Compute the shortest distances between attractors on the grid.

The presented methods for exploring paths and basins of attractions is of ‘stand-alone’ nature—they would also apply when the landscape is determined by alternative means such as stochastic simulations.

The method is based upon the mean field approximation in spin physics, where symmetric interactions are assumed. This is seldom the case in biological networks. We therefore introduced an approximation, whereby this mean field approximation is used for each degree of freedom separately. The Dijkstra algorithm is used for navigating in the resulting landscape. The latter is static once the interactions have been prescribed. Transition between the states or attractors occurs either with external manipulations as in the case of reprogramming experiments or spontaneously with spontaneous dynamics provided by low copy numbers [6,7].

Of note, the static landscape notion dealt with here is in concordance with the original Waddington picture. The distinction between this picture and the one with changes around the states as development proceeds was nicely elucidated in [1]. We agree with the view that network interaction changes lead to landscape modifications. However, such changes do not have to be as dramatic as in [1] and they do not rule out the possibility that decisions are made through stochastic transitions between existing states.

The notion that entropy or noise reflects an elevation in an energy landscape was discussed in [40] in connection with genome-wide analysis of putative networks in cancer cells.

Our approach offers possible recipes for optimal cell reprogramming strategies and proposes plausible scenarios for dynamics of stem cell commitment towards various lineages. The calculation of the shortest path from one stable attractor, that envisages a somatic cell state, to another attractor, linked to the pluripotent state, suggests that sequential steps should be considered for improving reprogramming efficiency. This striking result is confirmed by experimental studies reporting that sequential introduction protocol of Yamanaka [20] reprogramming factors (OCT4-Klf4 first, then c-MYC and finally SOX2) outperforms the simultaneous one [41]. It was also shown that reprogramming to iPSCs is a stepwise process [42] and furthermore critically dependent on the stage-specific control and action of all four transcription factors and Wnt signalling [43], where the latter switches from negative to positive regulation during reprogramming.

At first sight, one might question whether optimal reprogramming paths are given by the shortest ones in the free energy landscape. However, taking the dynamical approach, rate equations and free energy surfaces, at face value assuming all relevant components are in place, is the only option at hand. With noise present, the situation might be different on an event-by-event basis. However, averaging over these events makes sure that the deterministic approach provided here continues to be relevant.

In summary, we have provided means to exploit the landscape metaphor beyond being just an analogy. It is a useful framework to find optimal paths between stable states, thereby avoiding tedious trial-and-error procedures for rate equation parameters in reprogramming scenarios. This is particularly relevant for high-dimensional systems.

## 4. Methods

### 4.1. The free energy

The formalism behind equations (2.1)–(2.10) originates from Ising spin models in physics, where the degrees of freedom related to local magnetism take binary values *s*_*i*_=0,1, with an energy

4.1 $$E(s)=-\frac{1}{2}\sum_{ij}(\omega_{ij}s_{i}s_{j}+l_{i}s_{i}).$$

If this system is subject to fluctuations (or noise) described by a temperature *T*, it becomes stochastic obeying a Boltzmann distribution with probability for being in a state *s* given by

4.2 $$P(s)∼\exp\left(-\frac{E(s)}{T}\right).$$

Finding properties like steady states of the system then requires Monte Carlo simulations. In the mean field approximation, the stochastic dynamics of such systems can under certain conditions be approximated by deterministic equations as in equation (2.10) by minimizing equation (2.1) while replacing *s*_*i*_ in equation (4.1) with *v*_*i*_ thereby obtaining equation (2.9). The variables *v*_*i*_ then correspond to thermal averages of the original binary variables as *v*_*i*_=〈*s*_*i*_〉_*T*_ [44]. Different noise levels corresponding to different values of *T* give rise to different gain strengths in the sigmoids in equation (2.10). As mentioned above (Results), in the noiseless limit (T→0) for equations (2.1) and (2.10), a binary system is obtained. The concept of noise given by *T* is of course no different when applying the formalism to biological systems. The relationship between binary and analogue spin systems was already recognized in connection with electronic realizations of the Hopfield memory model [45]. A binary model was employed for similar purposes as ours in [6] with interaction strengths given by experimental correlations.

The mean field approximation assumes symmetric couplings, which is very seldom the case in transcriptional networks. Corrections can be derived [46] leading to extra terms in the argument of equation (2.5), the size of which depends upon the coupling strengths. However, in that case no free energy is defined. We have therefore chosen another route to circumvent the problem, namely to initially consider the free energy for each gene separately while the others are absorbed into ‘effective’ interaction strengths. These decomposed free energies are then merged to produce the final one. In practice, one starts from the rate equations determined by the architectures and integrate these one by one. It should be noted that this simplification does not reduce the procedure to something trivial as it is the mean field approach for each gene that connects the entropies with sigmoids.

Equation (2.9) can be easily generalized to several incoming genes. For example, in a three-gene system with two incoming genes *j* and *k* acting upon a third gene *i*, the generic energy is

4.3 $$E(v)=-\frac{1}{2}\sum_{ijk}({\tilde{\omega }}_{ijk}v_{i}v_{j}v_{k}+\omega_{ij}v_{i}v_{j}+\omega_{ik}v_{i}v_{k}+l_{i}),$$

with the entropy term (equation (2.3)) remaining the same and the corresponding steady-state equation for gene *i* is then given by

4.4 $$v_{i}=g\left(-\frac{1}{2T}\sum_{jk}(-{\tilde{\omega }}_{ijk}v_{j}v_{k}-\omega_{ij}v_{j}-\omega_{ik}v_{k}-l_{i})\right).$$

In equation (4.3), the first term represents the two incoming genes *j* and *k* acting cooperatively upon *i*, whereas the second and third term encode the corresponding independent actions.

We will limit ourselves to the independent action terms and in cases of multiple inputs encode the latter as effective genes in equation (4.3) thereby omitting the first term. This limitation is currently not unique for our sigmoidal approach but also present in Hill and Shea–Ackers formulations as the fittings otherwise become overly challenging. It typically requires exhaustive search among different options encoded and fitted separately (e.g. [47]).

In this context, one should mention an interesting approach for how to deal with cooperative interactions in transcriptional regulation whereby effective single genes are formed through feed-forward artificial neural network models [48].

The single-gene input interactions for the different systems we probe are found in electronic supplementary material, S1.

#### 4.1.1. Mapping the free energy landscape onto a directional graph

In order to explore the free energy landscape in an efficient manner we first discretize the expression values in each dimension (all *N* genes) varying in the interval [0,1] into *d*-positive values, thereby generating an *N*-dimensional grid for the free energy *F*. The latter is calculated from equation (2.1) for the resolution given by *d*. If needed, *F* is increased additively in order to ensure *F*≥0. The free energy difference between two adjacent configurations *v* and *u* is given by Δ*F*=*F*_**v**_−*F*_**u**_. If

4.5 ΔF>0

then an arrow points from *v* to *u* and *vice versa*. In this way, a directional network (not to be confused with the underlying biochemical network) between gene expression values is generated. For Δ*F*=0, an arbitrarily small value is added to one of the free energies to break the symmetry. In graph theory language, the nodes and arrows are called vertices and edges, respectively. The computational complexity of the steps involved in mapping out the arrows is $O(d^{N})$.

#### 4.1.2. Identifying attractors in landscapes

Cell states or attractors in biochemical networks are normally identified as steady states in the rate equations obtained from somewhat arbitrarily chosen different starting points. With access to the free energy landscape, it is possible to solidly identify all cell states. Given these states, different paths between them can then be investigated through the edges of the graph.

To this end, we first introduce the concept of strongly connected component (SCC), which is a direct subgraph such that for each pair of vertices (**v** and **u**) in the graph, there is a path from **v** to **u** and *vice versa*. Each SCC, which is identified by the Tarjan algorithm [49], can be reduced into a single vertex. One is then left with a reduced graph, in which an attractor or a cell state is detected as a vertex with no exiting edges and at least one entering edge.

A basin of attraction for an attractor is a set of initial conditions for the set of genes that brings only to that attractor. For each vertex in the reduced direct graph, one computes the shortest path to reach the attractor. This is done by calculating the sums of edges in each pathway, with non-negative lengths, with respect to the free energy changes Δ*F* satisfying the conditions of Dijkstra algorithm [5] of computational complexity O(number of edges×Nlog⁡(d)).

## Supplementary Material

Supporting Information S1

## Data accessibility

The electronic supplementary materials contain all formulae, parameters, procedures for reproducing the paper.
