# Supplementary material for: A deterministic method for estimating free energy genetic network landscapes with applications to cell commitment and reprogramming paths
Source: R Soc Open Sci. 2017 Jun 7;4(6):160765. doi: 10.1098/rsos.160765 (PMC5493897; doi:10.1098/rsos.160765)
Supplement: Supporting Information S1 [file rsos160765supp1.pdf]

Supporting Information S1

A deterministic method for estimating free energy genetic network landscapes with applications to cell commitment and reprogramming paths

Victor Olariu, Erica Manesso and Carsten Peterson

Free energies and dynamical equations

Below we provide the free energies formed out of single-gene decompositions corresponding to the rate equations together with parameter values for the five different networks employed for exploring the method. The "active" genes in energy function terms can be read off from the rate equations. For the synthetic single-gene switch we also perform a sensitivity analysis.

As discussed in the main text, we limit ourselves to single input action terms and in cases of multiple inputs encode the latter as effective genes (complexes).

The last term in Eq. 8 (main text) represents degradation. If multiplied with a degradation constant k_i, the latter would set the maximum expression of v_i - a rescaling. We have included a common parameter k in our examples, which turn out to be relevant only for the sensitivity analysis of the synthetic single-gene switch.

The attractor locations obtained from mapping out the free energies are in all cases verified by iterating the corresponding dynamical equations.

1. The synthetic single-gene motif

The free energy representing this system (Figure 2A) is given by

$$F(X;T) = -\frac{1}{2}\left(\omega_{X,X}X^2\right) + \frac{1}{2}\left(l_X X\right) + \frac{T}{2}\left[X \log X + (1-X)\log(1-X)\right] \tag{S1}$$

where X is the expression level of gene X. The corresponding dynamical equation is given by

$$\frac{dX}{dt} = g\left(-\frac{1}{2T}(-2\omega_{X,X}X - l_X)\right) - kX \tag{S2}$$

Here k is the degradation rate. Parameters values are found below in Table S1.

Table S1: Parameter values for the synthetic single-gene motif (Figure 2A).

Parameter	Process	Value
$\omega_{X,X}$	self-activation of X	1.50
l_X	external signal on X	1.50
k	degradation	1.00
T_{low}	low temperature	0.10
T_{high}	high temperature	0.50

Sensitivity Analysis

We conducted a simple sensitivity analysis for the synthetic single-gene switch. First, we calculated the free energy for the parameters values in Table S1 with the T_{high} value (blue lines in Figure S1). The magenta lines in Figure S1 show how variations of the self interaction strength $\omega_{X,X}$ affect the free energy landscape leading to modifications of the two attractors heights. When the self interaction strength is increased the high concentration attractors is most affected while when it is decrease the low concentration attractor height increases. The opposite effect was obtained when we varied the external signal strength l_X (orange lines in figure below). We also varied the decay rate k and observed major modification of the free energy as shown in Figure S1 with green lines. The sensitivity analysis shows that decay rates, self interactions and external signal strength have a major impact on the free energy landscape and slight variations of these parameters are able to influence cell fate.

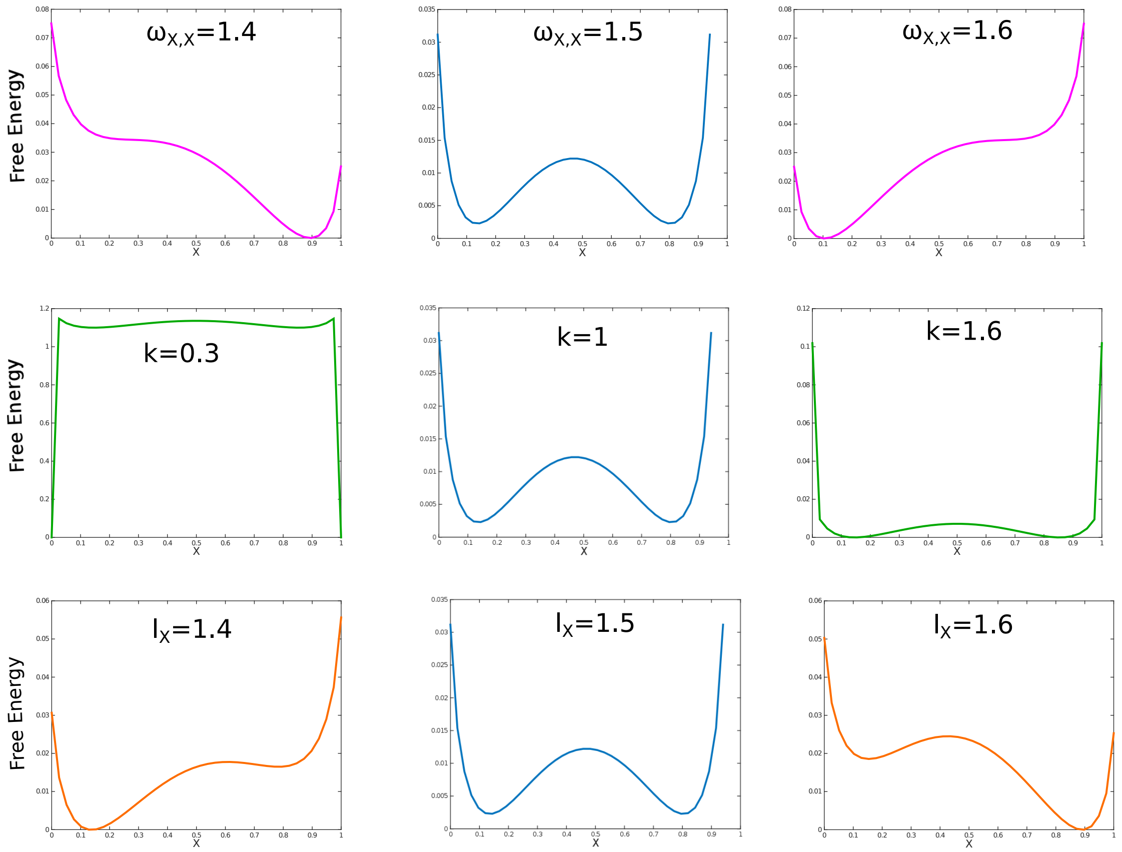

Figure S1: Sensitivity analysis for the synthetic single-gene switch. The free energy when the self interaction strength $\omega_{X,X}$ is varied (magenta), decay rate k is varied (green) and the external signal strength l_X is varied (orange). The blue curves show the free energy for the parameters values in Table S1. All graphs are for T_{high}.

2. The synthetic two-gene mutual repressor motif

The free energy representing this system (Figure 2C) is given by

$$F(X,Y;T) = -\frac{1}{2}(\omega_{XX}X^2 + \omega_{YX}XY + \omega_{XY}XY + \omega_{YY}Y^2) - \frac{1}{2}(l_X X + l_Y Y) + \frac{T}{2}[XlogX + (1-X)log(1-X) + YlogY + (1-Y)log(1-Y)] \quad \text{(S3)}$$

The corresponding dynamical equations are given by

$$\frac{dX}{dt} = g\left(-\frac{1}{2T}(-2\omega_{XX}X - \omega_{YX}Y + l_X)\right) - kX \quad \text{(S4)}$$

$$\frac{dY}{dt} = g\left(-\frac{1}{2T}(-2\omega_{YY}Y - \omega_{XY}X + l_Y)\right) - kY \quad \text{(S5)}$$

assuming the same degradation rate k for both genes. X and Y are the expression levels of genes X and Y respectively. Parameter values are found below in Table S2.

Table S2: Parameter values for the synthetic two-gene mutual repressor motif (Figure 2C).

Parameter	Description	Value
$\omega_{X,X}$	self-activation of X	4.00
$\omega_{Y,X}$	Y inhibition of X	-5.00
$\omega_{X,Y}$	X inhibition of Y	-5.00
$\omega_{Y,Y}$	self-activation of Y	4.00
l_X	external signal on X	1.00
l_Y	external signal on Y	1.00
k	degradation	1.00
T_{low}	low temperature	1.00
T_{high}	high temperature	2.00

3. The Gata2-Gfi1-Gfi1b regulatory motif

The hematopoietic gene regulatory network topology shown in Figure 3A was first introduced in [1]. We provide our corresponding free energy model with its associated sigmoidal rate equations. All links in the network are described by single parameters. Activations and repressions correspond to positive and negative parameter values respectively. The free energy representing the hematopoietic motif in Figure 3A is then given by:

$$\begin{aligned} F(G2, G1, Gb; T) = &-\frac{1}{2}\left(\omega_{G2,G2}G2^2 + \omega_{G1,G2}G2G1 + \omega_{G1,G1}G1^2 + \omega_{Gb,G1}G1^2Gb\right) \\ &-\frac{1}{2}\left(\omega_{Gb,Gb}Gb^2 + \omega_{G2,Gb}G2Gb + \omega_{G1,Gb}G1Gb^2\right) - \frac{1}{2}l_{G1}G1 \\ &+\frac{T}{2}\left[G2\log G2 + (1-G2)\log(1-G2) + G1\log G1 + (1-G1)\log(1-G1)\right] \\ &+\frac{T}{2}\left[Gb\log Gb + (1-Gb)\log(1-Gb)\right] \end{aligned} \tag{S6}$$

where $G2$, $G1$, and Gb are the expression levels of Gata2, Gfi1, and Gfi1b respectively. The corresponding dynamical equations are given by

$$\frac{dG2}{dt} = g\left(-\frac{1}{2T}(-2\omega_{G2,G2}G2 - \omega_{G1,G2}G1)\right) - kG2 \tag{S7}$$

$$\frac{dG1}{dt} = g\left(-\frac{1}{2T}(-2\omega_{G1,G1}G1 - \omega_{Gb,G1}G1Gb + l_{G1})\right) - kG1 \tag{S8}$$

$$\frac{dGb}{dt} = g\left(-\frac{1}{2T}(-2\omega_{Gb,Gb}Gb - \omega_{G1,Gb}G1Gb - \omega_{G2,Gb}G2)\right) - kGb \tag{S9}$$

assuming the same degradation rate k for Gata2, Gfi1, and Gfi1b. Parameter values are found below in Table S3.

Table S3: Parameter values for The Gata2-Gfi1-Gfi1b regulatory motif (Figure 2A).

Parameter	Description	Value
$\omega_{G2,G2}$	self-repression of Gata2	-0.05
$\omega_{G1,G2}$	Gfi1 repression of Gata2	-7.50
$\omega_{G1,G1}$	self-repression of Gfi1	-0.07
$\omega_{Gb,G1}$	Gfi1b repression of Gfi1	-18.0
$\omega_{G2,Gb}$	Gata2 activation of Gfi1b	10.0
$\omega_{G1,Gb}$	Gfi1 repression of Gfi1b	-0.80
$\omega_{Gb,Gb}$	self-activation of Gfi1b	0.05
l_{G1}	external signal on Gfi1	8.00
k	degradation	1.00
T	temperature	1.50

4. The Gata2-Gata1-Pu.1 regulatory motif

The hematopoietic motif shown in Figure 4A was first introduced in [2]. We provide our corresponding free energy model with its associated sigmoidal rate equations. All links in the network are described by single parameters. Activations and repressions correspond to positive and negative parameter values respectively. The free energy representing the hematopoietic motif in Figure 4A is then given by:

$$\begin{aligned} F(G2, G1, P1; T) = &-\frac{1}{2}\left(\omega_{G2,G2}G2^2 + \omega_{G1,G2}G2G1 + \omega_{P1,G2}G2P1 + \omega_{G1,G1}G1^2 + \omega_{G2,G1}G2G1\right) \\ &-\frac{1}{2}\left(\omega_{P1,G1}G1^2P1 + \omega_{P1,P1}P1^2 + \omega_{G2,P1}G2P1 + \omega_{G1,P1}G1P1^2\right) \\ &+\frac{1}{2}(l_{G2}G2 + l_{G1}G1 + l_{G1,EPO}G1EPO + l_{P1}P1) \\ &+\frac{T}{2}\left[G2\log G2 + (1-G2)\log(1-G2) + G1\log G1 + (1-G1)\log(1-G1)\right] \\ &+\frac{T}{2}\left[P1\log P1 + (1-P1)\log(1-P1)\right] \end{aligned} \tag{S10}$$

where $G2$, $G1$, and $P1$ are the expression levels of Gata2, Gata1, and Pu.1 respectively and EPO the Erythropoietin external signal. The corresponding dynamical equations are given by

$$\frac{dG2}{dt} = g\left(-\frac{1}{2T}(-2\omega_{G2,G2}G2 - \omega_{G1,G2}G1 - \omega_{P1,G2}P1 + l_{G2})\right) - kG2 \tag{S11}$$

$$\frac{dG1}{dt} = g\left(-\frac{1}{2T}(-2\omega_{G1,G1}G1 - \omega_{G2,G1}G2 - 2\omega_{P1,G1}G1P1 + l_{G1} + l_{G1,EPO}EPO)\right) - kG1 \tag{S12}$$

$$\frac{dP1}{dt} = g\left(-\frac{1}{2T}(-2\omega_{P1,P1}P1 - \omega_{G2,P1}G2 - \omega_{G1,P1}G1P1 + l_{P1})\right) - kP1 \tag{S13}$$

assuming the same degradation rate k for Gata2, Gata1, and Pu.1. Parameter values are found below in Table S4.

Table S4: Parameter values for the Gata2-Gata1-Pu.1 motif (Figure 3A).

Parameter	Process	Value
$\omega_{G2,G2}$	self-repression of Gata2	-5.00
$\omega_{G1,G2}$	Gata1 repression of Gata2	-0.67
$\omega_{P1,G2}$	Pu.1 repression of Gata2	-0.15
$\omega_{G1,G1}$	self-activation of Gata1	1.20
$\omega_{G2,G1}$	Gata2 activation of Gata1	0.26
$\omega_{P1,G1}$	Pu.1 repression of Gata1	-0.27
$\omega_{P1,P1}$	self-activation of Pu.1	7.30
$\omega_{G2,P1}$	Gata2 repression of Pu.1	-14.00
$\omega_{G1,P1}$	Gata1 repression of Pu.1	-24.00
$l_{G1,EPO}$	external signal on Gata1	0.30
EPO	external signal	25.00
l_{G2}	basal production of Gata2	0.53
l_{G1}	basal production of Gata1	0.52
l_{P1}	basal production of Pu.1	0.48
k	degradation	1.00
T	temperature	1.50

5. The Oct4/Sox2-Nanog-Fgf4-G regulatory motif

The topology of the gene regulatory network in Figure 5A is retrieved from [3] to which we added the experimentally proven Nanog self-repression [4]. We provide our corresponding free energy model with its associated sigmoidal rate equations. All links in the network are described by single parameters. Activations and repressions correspond to positive and negative parameter values respectively. The free energy representing the embryonic stem cell motif in Figure 5A is then given by:

$$\begin{aligned}
F(N,O,F,G;T) = &-\frac{1}{2}\left(\omega_{N,N}N^3O + \omega_{O,N}NO^2 + \omega_{F,N}NOF^2 + \omega_{G,N}NOG^2\right) \\
&-\frac{1}{2}\left(\omega_{O,O}O^3 + \omega_{G,O}OG^2 + \omega_{O,F}OF + \omega_{N,G}N^2G + \omega_{O,G}OG + \omega_{G,G}G^3\right) \\
&+\frac{1}{2}\left[\left(l_N + l_{N,L}LO\right)N + l_O O + \left(l_F + l_{F,I}I\right)F + l_G G\right] \\
&-\frac{T}{2}\left[N\log N + (1-N)\log(1-N) + O\log O + (1-O)\log(1-O)\right] \\
&-\frac{T}{2}\left[F\log F + (1-F)\log(1-F) + G\log G + (1-G)\log(1-G)\right] \qquad \text{(S14)}
\end{aligned}$$

where N, O, F, and G are the expression levels of Nanog, Oct4-Sox2, Fgf4-Gsk3 and G respectively and L and I are the external signals Lif-Bmp4 and 2i-3i. The corresponding dynamical equations are given by

$$\frac{dN}{dt} = g\left(-\frac{1}{2T}(-3\omega_{N,N}N^2O - \omega_{O,N}O^2 - \omega_{F,N}OF^2 - \omega_{G,N}OG^2 + l_N + l_{N,L}LO)\right) - kN \qquad \text{(S15)}$$

$$\frac{dO}{dt} = g\left(-\frac{1}{2T}(-3\omega_{O,O}O^2 - \omega_{G,O}G^2 + l_O)\right) - kO \qquad \text{(S16)}$$

$$\frac{dF}{dt} = g\left(-\frac{1}{2T}(-3\omega_{O,F}O + l_F + l_{F,I}I)\right) - kF \qquad \text{(S17)}$$

$$\frac{dG}{dt} = g\left(-\frac{1}{2T}(-3\omega_{G,G}G^2 - \omega_{N,G}N^2 - \omega_{G,O}O + l_G)\right) - kG \qquad \text{(S18)}$$

assuming the same degradation rate k for Nanog, Oct4-Sox2, Fgf4-Gsk3, and G. Parameter values are found below in Table S5.

Table S5: Parameter values for the Oct4/Sox2-Nanog-Fgf4-G regulatory motif (Figure 4A).

Parameter	Process	Value
$\omega_{N,N}$	self-repression of Nanog	-3.10
$\omega_{O,N}$	Oct4-Sox2 activation of Nanog	2.70
$\omega_{F,N}$	Fgf4-Gsk3 repression of Nanog	-0.10
$\omega_{G,N}$	G repression of Nanog	-2.10
$\omega_{O,O}$	self-activation of Oct4-Sox2	0.10
$\omega_{G,O}$	G repression of Oct4-Sox2	-7.00
$\omega_{O,F}$	Oct4-Sox2 activation of Fgf4-Gsk3	1.50
$\omega_{N,G}$	Nanog repression of G	-10.50
$\omega_{O,G}$	Oct4-Sox2 activation of G	1.50
$\omega_{G,G}$	self-activation of G	15.90
$l_{N,L}$	Lif-Bmp4 activation of Nanog	3.20
$l_{F,I}$	2i-3i repression of Fgf4-Gsk3	0.00
l_O	basal production of Oct4-Sox2	0.10
l_F	basal production of Fgf4-Gsk3	1.00
l_N	basal production of Nanog	1.00
l_G	basal production of G	1.00
k	degradation	1.00
T	temperature	0.17

References

[1] Moignard V *et al.* (2013) Characterization of transcriptional networks in blood stem and progenitor cells using high-throughput single-cell gene expression analysis. *Nat Cell Biol* 15:363-72.

[2] May G *et al.* (2013) Dynamic analysis of gene expression and genome wide transcription factor binding during lineage-specification of multipotent progenitors. *Cell Stem Cell* 6:754-768.

[3] Chickarmane V, Olariu V, Peterson C (2012) Probing the role of stochasticity in a model of the embryonic stem cell: heterogeneous gene expression and reprogramming efficiency. *BMC Syst Biol* 6:98.

[4] Navarro P *et al.* (2012) OCT4/SOX2-independent Nanog autorepression modulates heterogeneous Nanog gene expression in mouse ES cells. *EMBO J.* **31,** 4547-4562.
